# Dietary supplementation with microbially fermented rice bran promotes lactation performance in dairy cows by increasing rumen fermentation performance and nutrient digestibility

**DOI:** 10.3389/fvets.2025.1713279

**Published:** 2026-02-20

**Authors:** Zixiao Zhang, Wanhao Cai, Xiaoshi Wei, Bo He, Yanze Liu, Xiaowei Zhang, Jinyong Yang, Fusheng Li, Zhefeng Li, Chong Wang

**Affiliations:** 1 Modern Farming (Group) Co., Ltd, Maanshan, China; 2 Key Laboratory of Applied Technology on Green-Eco-Healthy Animal Husbandry of Zhejiang Province, College of Animal Science and Technology & College of Veterinary Medicine of Zhejiang A&F University, Hangzhou, China; 3 Yueqing Livestock and Veterinary Development Center, Yueqing, China; 4 Zhejiang Provincial Animal Husbandry Technology Promotion and Monitoring Station of Breeding Livestock and Poultry, Hangzhou, China; 5 Inner Mongolia Baihe Biotechnology Co., Ltd, Huhehaote, China; 6 Hangzhou King Techina Feed Co., Ltd., Hangzhou, Zhejiang, China

**Keywords:** dairy cow, lactation performance, microbially fermented rice bran feed, milk composition, rumen microbial community

## Abstract

**Introduction:**

Microbial fermentation effectively addresses the issue of rice bran rancidity also enhances its nutritional value as animal feed. This study aimed to explore the effects of dietary microbially fermented rice bran feed (MFRB) supplementation on lactation performance, nutrient digestibility, plasma biochemical indicators, rumen fermentation parameters and microbiota of lactating dairy cows.

**Methods:**

Thirty Holstein cows with similar milk yield (38.1 ± 1.0 kg/d), days in milk (282.8 ± 2.2 d) and parity (2.37 ± 0.1) were randomized into two groups: (1) CON (Control group, fed a basal diet); (2) MFRB (2.6% of pelleted corn was replaced with MFRB). The experiment consisted of a 7-day adaptation period followed by a 30-day experimental period.

**Results:**

As a result, despite a lower dry matter intake, dairy cows fed MFRB achieved significantly higher milk yield, feed efficiency, milk fat yield, milk protein percentage, fat-corrected milk, and energy-corrected milk (*P* < 0.05). Apparent neutral detergent fiber digestibility was also significantly increased (P < 0.05), with trends toward increased dry matter and crude protein digestibility (0.05 < *P* < 0.1). Rumen fermentation shifted to a propionate-dominant pattern, with significantly higher levels of propionate, ammonia-N, microbial protein, and Prevotella abundance (*P* < 0.05). plasma triglycerides and glucose levels were increased, while alanine aminotransferase and aspartate aminotransferase activities decreased (*P* < 0.05).

**Discussion:**

In conclusion, these integrated changes demonstrate that MFRB enhanced rumen fermentation performance, nutrient utilization, and metabolic health, ultimately optimizing lactational performance.

## 1 Introduction

According to the Food and Agriculture Organization (FAO) of the United Nations, approximately 86% of livestock feed is derived from materials inedible by human consumption, including crop residues and agricultural by-products. This high level of utilization underscores the pivotal role of the livestock sector in upcycling agricultural waste (https://www.cgiar.org/news-events/news/fao-sets-the-record-straight-86-of-livestock-feed-is-inedible-by-humans). The effective reuse of these materials not only mitigates the competition between food and feed resources but also enhances resource efficiency and sustainability, thereby contributing to the realization of circular economy goals (1, 2).

As a major by-product of rice processing, rice bran has an annual global output of approximately 68 million tons (3). While a small portion is processed to extract rice bran oil (4), the majority is subjected to low-value uses such as composting or incineration, resulting in significant resource waste and environmental pollution (5). Moreover, rich bran is rich in high-quality nutrients, including lipids, proteins, and dietary fibers, and also contains abundant unsaturated fatty acids and fermentable fibers, making it a promising candidate for high-value feed applications (6). However, studies have shown that its high content of unsaturated fatty acids is prone to rapid degradation due to lipase activity, leading to a decline in nutritional quality (7). Although mechanical processing and heat treatment technologies can extend its shelf life, these methods often require substantial energy input and infrastructure, raising processing costs (8, 9). In contrast, microbial fermentation offers a promising low-cost approach to the efficient utilization of rice bran.

Microbially fermented rice bran feed (MFRB) has the potential to convert this low-value by-product into a nutritionally rich and stable feed ingredient. Through microbial fermentation, the crude protein (CP) and fat contents of rice bran can be significantly enhanced, while its fiber and phytic acid levels are reduced, thereby improving its nutritional profile and digestibility. These improvements have made MFRB particularly suitable for incorporation into non-ruminant diets (10, 11). Najoan et al. (12) used *Aspergillus niger* for solid-state fermentation of rice bran and showed that including 40% MFRB in dairy cattle rations improved feed efficiency (FE) and lactation performance. Moreover, anaerobic fermentation of rice bran using rumen microbes significantly decreased crude fiber and NDF and released inorganic phosphorus, suggesting improved digestibility in ruminants (13). Repeated feeding experiments across species have confirmed the benefits of MFRB in animal production. In laying hens, inclusion of 2.5–5.0% MFRB increased average daily feed intake, improved digestibility of dry matter (DMD), digestibility of CP (CPD) and digestibility of crude fiber, and enriched cecal populations of SCFA-producing microbes (14). In Hu sheep, replacing 15% of soybean straw with MFRB hulls significantly increased average daily gain, enhanced CPD, and altered rumen fermentation profiles including butyrate, valerate, and microbial diversity (15). In pigs, liquid fermented feed using alternative substrates led to increased feed intake, weight gain, nutrient digestibility, proliferation of *Lactobacillus* in the gut, and elevated volatile fatty acid (VFA) levels (16). Although not directly using rice bran, *in vitro* MFRB with *Lactobacillus marei* also improved rumen fermentation characteristics, supporting its potential *in vivo* application (17). Therefore, MFRB demonstrates substantial promise in improving nutrient utilization, animal performance, gut health, and fermentation efficiency across various species, offering strong theoretical and empirical support for its incorporation into dairy cows.

This study aimed to comprehensively evaluate the feasibility of MFRB in lactating Holstein cows, focusing on its effects on lactation performance, nutrient apparent digestibility, plasma biochemical indicators, rumen fermentation parameters and microbial community. Through a multifaceted analysis ranging from production performance to lactation-related mechanisms, this study was designed to clarify the role of MFRB in the nutritional regulation of dairy cows and to provide a solid theoretical basis and practical guidance for optimizing dairy cow feeding strategies and improving milk production and quality.

## 2 Materials and methods

### 2.1 Animal ethics statement

All experimental procedures were approved by the Animal Protection and Utilization Committee of Zhejiang Agriculture and Forestry University, Hangzhou, China (ZAFUAC202433) and were in accordance with the university's animal research rules. All experimental animals, design, and animal management were conducted in compliance with the “Animal Research: Reporting of *In Vivo* Experiments” (ARRIVE) guidelines (https://arriveguidelines.org).

### 2.2 Animals, diets and experimental design

The MFRB used in this experiment was produced as follows: the rice bran was taken as the main substrate, inoculated with beneficial microbes (*Bacillus licheniformis, Bacillus subtilis, Lactobacillus acidophilus* and *Saccharomyces cerevisiae*), and fermented under anaerobic conditions at ambient temperatures above 25 °C for 10 days. The chemical composition of the MFRB was as follows: DM 40%−60%, CP ≥ 10%, crude fiber ≤ 25%, crude ash (Ash) ≤ 15%, phosphorus ≥ 0.4%, calcium 0.3%−2.5%, sodium chloride 0.3%−1.0%.

Based on similar parity, milk yield, and days in milk (DIM), two groups of 30 Holstein lactating dairy cows were assigned to a control group (CON; parity 2.4 ± 0.1, DIM 280.1 ± 3.2 d, milk yield 39.2 ± 1.6 kg/d) and a treatment group (MFRB; parity 2.3 ± 0.13, DIM 284.9 ± 2.9 d, milk yield 39.7 ± 1.7 kg/d). The cows in the CON group were fed the basal diet, and the cows in the MFRB group were fed the experimental diet, which was adjusted according to isoenergetic and isonitrogenous principles by replacing 5.3% of pressed flaked maize in the basal diet with 2.6% MFRB on a dry matter basis (Table 1). The two diets were formulated according to NRC (18) guidelines at a 60:40 concentrate-to-forage ratio to satisfy minimum energy, protein, mineral, and vitamin requirements, and offered as total mixed ration (TMR) twice a day. The cows were kept in two separate facilities, had free access to water all time, and milked three times per day. The experiment comprised a 7-day adaptation period followed by a 30-day experimental period.

**Table 1:** Ingredients and chemical composition of the experimental diets.

**Items, % of DM**	**Group** ^**1**^	
	**CON**	**MFRB**
**Ingredients**		
Soybean meal	15.12	15.59
Corn flour	11.60	11.96
Oat hay	4.54	4.68
Alfalfa hay	4.59	4.74
Corn silage	29.48	30.39
Molasses	3.51	3.62
RP soybean meal	3.44	3.55
Cottonseed	4.21	4.34
Rapeseed meal	1.15	1.18
DDGS	2.22	2.29
MgO	0.14	0.14
RP methionine	0.08	0.08
Active dry yeast	0.06	0.06
NaHCO3	1.06	1.10
Glucose powder	0.89	0.92
Fat powder	2.18	2.25
Premix^2^	2.41	2.48
Pelleted corn	13.32	8.01
MFRB^3^	0.00	2.62
**Chemical composition**		
DM	44.61	44.37
CP	17.36	17.59
NDF	29.82	29.31
ADF	17.03	16.78
Ash	7.52	7.36
Calcium	0.81	0.81
Phosphorus	0.41	0.40
${\text{NE}}_{\text{L}}^{4}$, Mcal/kg	1.66	1.66

RP, rumen protected; DDGS, distillers dried grains with soluble; MgO, magnesium oxide; NaHCO3, sodium bicarbonate; MFRB, microbially fermented rice bran feed; DM, dry matter; CP, crude protein; NDF, neutral detergent fiber; ADF, acid detergent fiber; NE_L_, net energy for lactation.

^1^Base on the isonitrogenous and isoenergetic principle, 0% (CON) and 2.6% (MFRB) of microbially fermented rice bran feed were used to replace pelleted corn in the basic diet (dry matter basis).

^2^The premix mix contains the following nutrients level per kilogram: Cu 250–400 mg, Fe 900–1,440 mg, Mn 680–1,100 mg, Zn 840–1,360 mg, vitamin A 90,000–196,000 IU, vitamin D3 35,000–77,000 IU, vitamin E 1,000 IU, moisture ≤ 9%.

^3^The chemical composition of microbial fermented feed is as follows: dry matter 40%-60%, crude protein ≥ 10%, crude fiber ≤ 25%, ash ≤ 15%, phosphorus ≥ 0.4%, calcium 0.3%-2.5%, sodium chloride 0.3%-1.0%.

^4^Net energy concentration of the diets was estimated using CPM CNCPS v3.0.8.1 (Cornell University, Ithaca, NY).

### 2.3 Sample collection and measurements

Throughout the experimental period, daily TMR offered and refused were recorded for each group to calculate average dry matter intake (DMI). Every 5 days, 500 g of fresh TMR was collected, for a total of six sampling events. TMR samples were used for DM and chemical analysis.

Milk yield was monitored daily via the SCR Data Flow II system (Allflex Livestock Intelligence, Beijing, China), and FE was calculated as the milk yield divided by the DMI. On 1 d, 10 d, 20 d, and 30 d, composite milk samples of 50 mL were collected from the milking in the morning, midday and evening milkings in a 4:3:3 ratio, preserved with potassium dichromate and stored at −4 °C for component analysis.

At the end of the experiment, eight dairy cows per group were randomly selected for fecal, plasma, and rumen fluid samples collection. On 27 d−29 d, rectal fecal specimens were collected four times per day over three consecutive days, yielding 12 samples per cow. Fecal samples were dried at 65 °C for 48 h for chemical analysis and digestibility calculations.

After 2 h of morning feeding on 30 d, blood was drawn from the coccygeal vein into heparin tube, centrifuged at 3,500 × *g* for 15 min at 4 °C, and plasma was stored at −80 °C for biochemical analyses.

Rumen fluid was collected via a rumen sampler: the first 30 mL were discarded to avoid saliva contamination, and the subsequent sample was filtered through four layers of gauze and stored at −80 °C for fermentation parameter and microbiota analyses.

### 2.4 Calculation and chemical analysis

The DM (method. 930.15), ether extract (EE; method. 920.39), CP (method. 991.20), Ash (method. 942.05), calcium (method. 968.08) and phosphorus (method. 965.17) in diet and feces were analyzed by AOAC methods (19). DM was determined by drying the samples at 105 °C for 3 h, and all chemical analyses were conducted based on the final absolute DM. EE was measured using the Soxhlet extraction method. Nitrogen content in feces and diet was determined by the Kjeldahl method, and CP was calculated as nitrogen × 6.25. Ash content was determined by combusting the samples at 550 °C for 4 h. Calcium content was analyzed using flame atomic absorption spectrophotometry, and total phosphorus content was determined by a colorimetric procedure. Neutral detergent fiber (NDF) and acid detergent fiber (ADF) were determined according to the procedure described by Van Soest et al. (20). Net energy concentration (NE_L_) of the diets was estimated using CPM CNCPS v3.0.8.1 (Cornell University, Ithaca, NY). Acid insoluble ash (AIA) content of feces and diet was analyzed according to the method of Van Keulen and Young (21).

All milk samples were analyzed using a MilkoScan infrared spectrometer (Foss Electric A/S, Hillerød, Denmark) to determine milk fat, protein, lactose and milk density. Fat corrected milk (FCM) and energy corrected milk (ECM) were calculated as Equations 1, 2, respectively:


$$\begin{array}{c} FCM\;\left(kg\right)=0.4\times milk\;fat\;yield+15\times milk\;yield \end{array}$$ (1)

$$\begin{array}{c} ECM\;\left(kg\right)=0.323\times milk\;fat\;yield+7.13\times milk\;protein\;yield \end{array}$$

$$\begin{array}{c} +12.82\times milk\;yield \end{array}$$ (2)

where milk fat yield (kg) and milk protein yield (kg) were calculated by milk yield (kg) multiply milk fat percentage (%) and multiply milk protein percentage (%), respectively.

The apparent digestibility of nutrients was determined by the AIA method, using indigestible AIA in the diet and fecal samples as an internal marker to calculate the apparent digestibility of each nutrient. The calculation was performed as Equation 3 (22):


$$\begin{array}{l} Digestibility(\%)=[1-(AIA_{diet}/AIA_{fecal})\\ \;\times (Nutrient_{fecal}/Nutrient_{diet})]\times 100 \end{array}$$ (3)

where *Nutrient*_*diet*_ (%) and *Nutrient*_*fecal*_ (%) are the nutrient contents, and *AIA*_*diet*_ (%) and *AIA*_*fecal*_ (%) are the AIA contents in the diets and fecal samples, respectively.

Plasma total protein, albumin, blood urea nitrogen (BUN), triglycerides, non-esterified fatty acids (NEFA), glucose, alkaline phosphatase (ALP), alanine aminotransferase (ALT), aspartate aminotransferase (AST), total bilirubin, beta-hydroxybutyrate (BHBA), and blood ketones were determined using commercial kits according to the manufacturer's instructions (Nanjing Jiancheng Bioengineering Institute, Nanjing, China).

The pH value of rumen fluid samples was measured using a digital pH meter (Ph818M, Hong Kong Smart Sensor Ltd., China). Samples for the determination of ammonia nitrogen (NH_3_-N) and microbial protein (MCP) were centrifuged at 3,000 × *g* for 15 min at 4 °C. NH_3_-N concentration was determined using the colorimetric method of Verdouw et al. (23), while MCP content was assessed via the purine method (24), both measurements being performed with a microplate reader (BioTek Powerwave XS, VT, USA). To 1 mL of rumen fluid, 200 μL of 25% metaphosphoric acid was added and mixed by manually vortexing. The mixture was centrifuged at 12,000 × *g* at 4 °C for 10 min. The supernatant was subjected to gas chromatography (Agilent 7890B, CA, USA) to determine the concentrations of VFA (25).

Total DNA of the rumen fluid samples were extracted by utilizing the E.Z.N.A.^®^ Soil DNA Kit (Omega Bio-Tek, Norcross, GA, USA). The V3-V4 regions of the bacterial 16S rRNA gene were amplified employing the forward primer 338F (ACTCCTACGGGAGGCAGCAG) and the reverse primer 806R (GGAC-TACHVGGGTWTCTAAT). The PCR products were quantified with the Quant-iTPicoGreen dsDNA Assay Kit (Invitrogen, Carlsbad, USA). The amplicon libraries were generated by pooling in an equal ratio and Sequencing was performed on Illumina MiSeq platform (Majorbio Bio-Pharm Technology Co., Ltd., Shanghai, China). Microbiota bioinformatics analysis was conducted using QIIME 2 (26). The DADA2 denoise-paired method was applied to process the sequences by quality filtering, denoising, merging, and removing chimeras. Amplicon sequence variants (ASVs) were clustered at 97% similarity using the Greengenes 16S reference database (gg_13).

### 2.5 Statistical analysis

Repeated-measures ANOVA and one-way ANOVA were performed using the “PROC MIXED” procedure in SAS software (version 9.4, SAS Institute Inc., Cary, NC, USA) for all variables except for DMI and rumen bacteria community data, following the models in Equations 4, 5, respectively:


$$\begin{array}{c} Y_{ijk}=\mu +M_{i}+D_{j}+{\left(T\times D\right)}_{ij}+A_{k\left(i\right)}+e_{ijk} \end{array}$$ (4)

where *Y*_*ijk*_ is the dependent variable, μ is the overall mean, *M*_*i*_ is the fixed effect of MFRB (*i* = 2, CON and MFRB), *D*_*j*_ is the fixed effect of day (*j* = 1 to 30 per lactation performance or and *j* = 1, 10, 20, and 30 for milk composition), *(T*×*D)*_*ij*_ is their interaction, *A*_*k*(*i*)_ is the random effect of cow_*k*_ nested within treatment (*k* = 15), and *e*_*ijk*_ is the residual error. *Post-hoc* comparisons of least-square means were performed using Tukey's honestly significant difference when main or interaction effects were significant (*P* < 0.05).


$$\begin{array}{c} Y_{ij}=\mu +M_{i}+A_{j}+e_{ij} \end{array}$$ (5)

where *Y*_*ij*_ is the dependent variable, μ is the overall mean, *M*_*i*_ is the fixed effect of MFRB (*i* = 2, CON and MFRB), *A*_*j*_ is the random effect of cows (*j* = 8 for nutrient apparent digestibility, plasma biochemical indicators and Rumen fermentation characteristics), and *e*_*ij*_ is the residual error.

Paired-samples *t*-test was used to statistical analysis daily DMI (*n* = 30; PROC TTEST), and Wilcoxon rank-sum test was used to compare rumen bacteria community differences (*n* = 8; PROC NPAR1WAY). Correlation analysis was performed using Spearman's correlation and Mantel-test. Data were expressed as least squares means, and differences were considered statistically significant at *P* < 0.05, with tendencies discussed at 0.05 ≤ *P* < 0.10.

## 3 Results

### 3.1 Lactation performance

**Figure 1:**
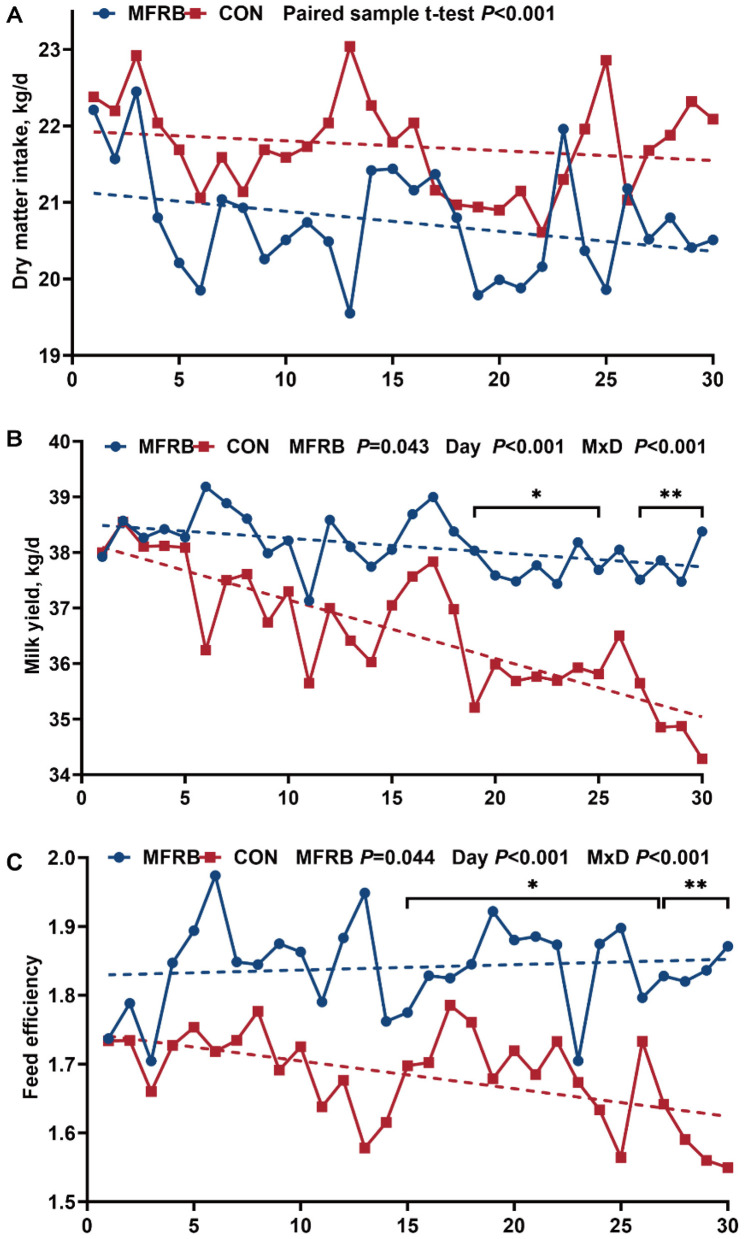
Production performance of lactating dairy cows. **(A)** Dry matter intake. **(B)** Milk yield. **(C)** Feed efficiency. Base on the isonitrogenous and isoenergetic principle, 0% (CON) and 2.6% (MFRB) of microbially fermented rice bran feed were used to replace pelleted corn in the basics diet (dry matter basis). The dashed line indicates the trend of the corresponding parameter. Main effects of treatment (MFRB) and day (Day) and their interaction (M × D) are shown on the plot. Main effects and their interaction were analyzed using repeated-measures ANOVA. *P < 0.05; **P < 0.01.

Figure 1 illustrated the effect of dietary supplementation with MFRB on lactation performance in lactating dairy cows. Compared with the CON group, the MFRB group showed a lower DMI (*P* < 0.05), averaging 20.71 kg/d vs. 21.73 kg/d, a reduction of 1.02 kg/d (Figure 1A). During the 30-day experimental period, milk yield and FE in the MFRB group were significantly higher than in the CON group as a whole (*P* < 0.05). Specifically, average milk yield in the MFRB group was 38.12 ± 1.28 kg/day, compared to 36.54 ± 1.21 kg/d in the CON group, showing an increase of 1.58 kg (Figure 1B). Similarly, the MFRB group achieved a higher average FE of 1.84 ± 0.06, vs. 1.68 ± 0.06 in the CON group, indicating an improvement of 0.16 units (Figure 1C). Further analysis showed that milk yield was significantly higher in the MFRB group than in the CON group during d 19–d 30. Likewise, FE was significantly higher in the MFRB group than in the CON group during d 15–d 27 (*P* < 0.05) and d 27- d 30 (*P* < 0.01). In addition, milk yield and FE also showed significant variation over time (*P* < 0.001) and a significant interaction between MFRB and day was observed (*P* < 0.001).

### 3.2 Milk composition

**Figure 2:**
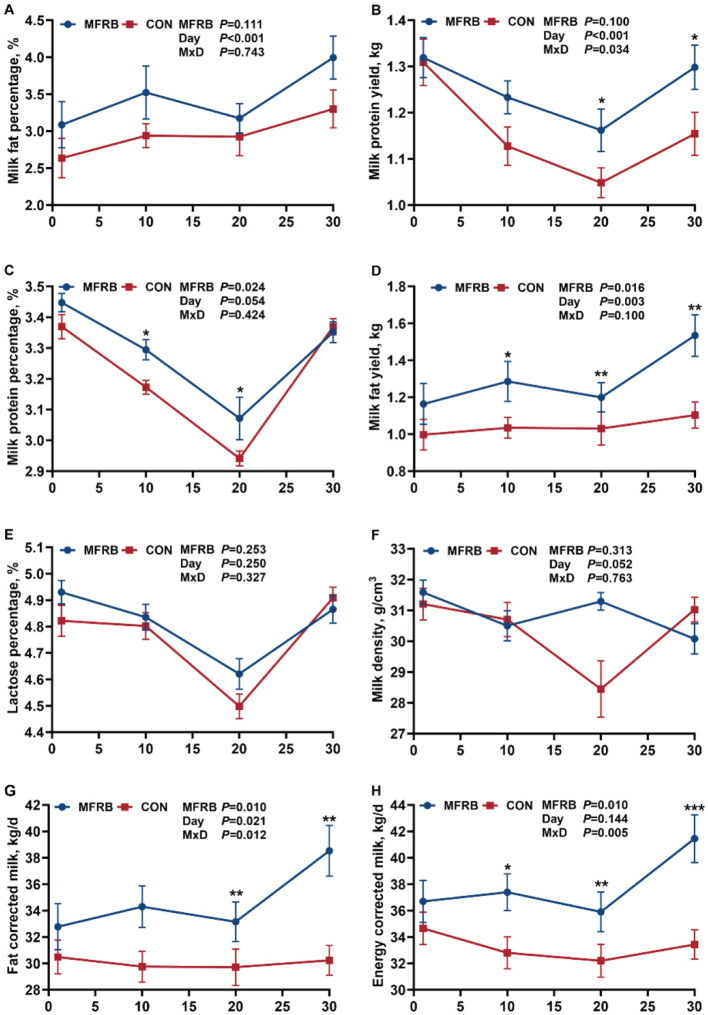
Milk composition of lactating dairy cows. **(A)** Milk fat percentage. **(B)** Milk fat yield. **(C)** Milk protein percentage. **(D)** Milk protein yield. **(E)** Lactose percentage. **(F)** Milk density. **(G)** Fat corrected milk. **(H)** Energy corrected milk. Base on the isonitrogenous and isoenergetic principle, 0% (CON) and 2.6% (MFRB) of microbially fermented rice bran feed were used to replace pelleted corn in the basics diet (dry matter basis). Main effects of treatment (MFRB) and day (Day) and their interaction (M × D) are shown on the plot. Main effects and their interaction were analyzed using repeated-measures ANOVA. *P < 0.05; **P < 0.01; ***P < 0.001.

Dietary supplementation with MFRB did not significantly increase the milk fat percentage (*P* = 0.111; Figure 2A), but did result in a significantly higher milk fat yield (*P* = 0.016; Figure 2B). Milk fat yield was significantly higher in the MFRB group than in the CON group at 10 d, 20 d, and 30 d (*P*<*0.05*). In addition, the addition of MFRB significantly increased the milk protein percentage (*P* = 0.024; Figure 2C), which was significantly higher in the MFRB group on 10 d and 20 d (*P* < 0.05). Although the overall difference in milk protein yield between the two treatment groups was not significant (*P* = 0.100; Figure 2D), it was influenced by a significant “Day effect” (*P* < 0.001) and a “MFRB × Day” interaction (*P* = 0.034). Further analysis revealed that milk protein yield was significantly higher in the MFRB group on 20 d and 30 d (*P* < 0.05). Both lactose percentage and milk density did not differ significantly between the MFRB and CON groups (*P* > 0.05; Figures 2E, F). Dietary supplementation with MFRB significantly increased both FCM and ECM in lactating cows (*P* < 0.05; Figures 2G, H). Specifically, FCM and ECM were significantly higher in the MFRB group compared to the CON group on 20 d and 30 d (*P* < 0.05), and ECM was also up-regulated in the MFRB group on 10 d (*P* < 0.05). Moreover, there was a significant “MFRB and days” interaction for FCM and ECM (*P* < 0.05).

### 3.3 Nutrient apparent digestibility

As shown in Table 2, dietary supplementation with MFRB had a significant effect on nutrient apparent digestibility of lactating dairy cows. MFRB significantly increased digestibility of NDF (NDFD) by 1.73% in lactating dairy cows (*P* = 0.028). In addition, DMD and CPD of dairy cows in the MFRB group also tended to be higher (0.05 < *P* < 0.1), but there was no significant difference in EE digestibility or ADF digestibility (*P* > 0.05).

**Table 2:** Nutrient apparent digestibility of lactating dairy cows.

**Items, %**	**Group** ^**1**^		**SEM**	***P*-value**
	**CON**	**MFRB**		
DM	75.74	76.68	0.346	0.054
CP	68.43	69.20	0.287	0.055
EE	71.77	72.22	0.328	0.243
NDF	50.48	52.21	0.513	0.028
ADF	45.40	45.51	0.617	0.859

DM, dry matter; CP, crude protein; EE, ether extract; NDF, neutral detergent fiber; ADF, acid detergent fiber.

^1^Base on the isonitrogenous and isoenergetic principle, 0% (CON) and 2.6% (MFRB) of microbially fermented rice bran feed were used to replace pelleted corn in the basics diet (dry matter basis).

### 3.4 Plasma biochemical indicators

Table 3 illustrated the effect of dietary supplementation with MFRB on plasma biochemical indicators in lactating dairy cows. Compared to the CON group, plasma triglyceride and glucose levels were significantly higher in the MFRB group (*P* < 0.05), in addition to a trend of significantly lower blood ketone levels in the MFRB group (0.05 < *P* < 0.1). Surprisingly, dietary supplementation with MFRB had a significant effect on plasma liver function indicators. Specifically, plasma ALT and AST levels were significantly lower while ALP levels were significantly higher in the MFRB group compared to the CON group (*P* < 0.05). There was no significant difference in plasma total protein, albumin, BUN, NEFA, total bilirubin and BHBA (*P* > 0.05).

**Table 3:** Plasma biochemical indicators of lactating dairy cows.

**Items**	**Group** ^**1**^		**SEM**	***P*-value**
	**CON**	**MFRB**		
Total protein, g/L	72.42	71.63	0.392	0.116
Albumin, g/L	34.59	33.99	0.415	0.219
BUN, mmol/L	5.23	5.21	0.012	0.184
Triglyceride, mmol/L	0.22	0.24	0.009	0.047
NEFA, mmol/L	30.50	30.73	0.511	0.680
Glucose, mmol/L	4.25	4.28	0.008	0.021
ALP, U/L	42.49	44.11	0.486	0.029
ALT, U/L	31.46	26.63	0.716	0.003
AST, U/L	74.22	72.50	0.571	0.039
Total bilirubin, μmol/L	4.94	4.94	0.013	0.815
BHBA, mmol/L	0.39	0.40	0.008	0.288
Blood ketone, mg/dL	2.01	1.54	0.183	0.063

BUN, blood urea nitrogen; NEFA, non-esterified fatty acid; ALP, alkaline phosphatase; ALT, alanine aminotransferase; AST, aspartate aminotransferase; BHBA, beta-hydroxybutyrate.

^1^Base on the isonitrogenous and isoenergetic principle, 0% (CON) and 2.6% (MFRB) of microbially fermented rice bran feed were used to replace pelleted corn in the basics diet (dry matter basis).

### 3.5 Rumen fermentation parameters

As shown in Table 4, dietary supplementation with MFRB had a significant effect on rumen fermentation parameters in lactating dairy cows, and the rumen concentrations of NH_3_-N and MCP were significantly higher in the MFRB group than in the CON group (*P* < 0.05), but there was no significant change in rumen pH value (*P* > 0.05). Regarding rumen VFA, dietary supplementation with MFRB significantly increased rumen propionate concentration (*P* < 0.05), which led to a significant increase in rumen total VFA (TVFA) concentration (*P* < 0.05) and a significant decrease in acetate-to-propionate ratio (A:P ratio) (*P* < 0.05). However, MFRB had no significant effect on the rumen concentrations of acetate and butyrate (*P* > 0.05).

**Table 4:** Rumen fermentation parameters of lactating dairy cows.

**Items**	**Group** ^**1**^		**SEM**	***P*-value**
	**CON**	**MFRB**		
pH	6.22	6.20	0.028	0.505
NH_3_-N, mg/dL	2.24	3.05	0.125	0.001
MCP, mg/mL	1.82	2.02	0.011	< 0.001
Total VFA, mmol/L	105.66	106.95	0.502	0.042
Acetate, mmol/L	61.68	61.78	0.309	0.775
Propionate, mmol/L	25.53	26.60	0.223	0.003
Butyrate, mmol/L	12.23	12.38	0.107	0.205
A:P ratio	2.42	2.32	0.017	0.002

NH_3_-N, ammonia-nitrogen; MCP, microbial crude protein; VFA, volatile fatty acids; A:P ratio, acetate-to-propionate ratio.

^1^ Base on the isonitrogenous and isoenergetic principle, 0% (CON) and 2.6% (MFRB) of microbially fermented rice bran feed were used to replace pelleted corn in the basics diet (dry matter basis).

### 3.6 Rumen microbial community composition

A total of 435,370 reads from 25,618 ASVs were observed in 16 fecal rumen fluid samples with an average of 27,210.6 ± 421.8 reads and 1,601.1 ± 39.8 ASVs per sample. Five dominant phyla and ten dominant genera consistently present in all rumen fluid samples were identified as shown in Table 5. At the phylum level, the predominant bacterial phyla in the rumen of lactating dairy cows were Bacteroidetes, Firmicutes, and Proteobacteria, collectively accounting for over 90% of the total bacterial abundance. However, there were no significant differences in the relative abundances of these three phyla between the MFRB and CON groups (*P* > 0.05). At the genus level, the dominant genera included *Prevotella, Ruminococcus, Rikenellaceae_RC9_gut_group, Christensenellaceae_R-7_group, and Ruminococcaceae_NK4A214_group*. According to the Wilcoxon rank-sum test, dietary supplementation with MFRB significantly increased the relative abundance of *Prevotella* in the rumen of lactating cows (*P* = 0.009), whereas no significant differences were observed for the other major genera between the two groups (*P* > 0.05).

**Table 5:** Relative abundance of dominant phylum and genus of rumen bacteria in lactating dairy cows.

**Items**	**Group** ^**1**^		**SEM**	***P*-value^2^**
	**CON**	**MFRB**		
**Phyla** ^3^ **, %**				
Bacteroidetes	52.23	52.53	0.177	0.149
Firmicutes	31.58	31.41	0.227	0.488
Proteobacteria	9.87	9.78	0.133	0.520
Fibrobacteres	3.96	3.86	0.097	0.313
Spirochaetes	2.07	2.05	0.117	0.864
others	0.29	0.39	0.093	0.359
**Genera** ^4^ **, %**				
*Prevotella*	21.01	21.92	0.235	0.009
*Ruminococcus*	8.11	7.93	0.275	0.543
*Rikenellaceae_RC9_gut_group*	6.42	6.00	0.250	0.144
*Christensenellaceae_R-7_group*	4.76	4.99	0.355	0.531
*Ruminococcaceae_NK4A214_group*	5.58	5.74	0.366	0.674
*Ruminococcaceae_UCG-014*	4.21	4.40	0.306	0.562
*Succinivibrionaceae_UCG-001*	4.93	5.34	0.302	0.229
*Succinivibrionaceae_UCG-002*	4.50	4.60	0.259	0.726
*Fibrobacter*	3.92	3.76	0.230	0.684
*Butyrivibrio*	3.65	2.99	0.485	0.223
*others*	32.91	32.33	0.905	0.396

^1^Base on the isonitrogenous and isoenergetic principle, 0% (CON) and 2.6% (MFRB) of microbially fermented rice bran feed were used to replace pelleted corn in the basics diet (dry matter basis).

^2^*P*-values were calculated using the Wilcoxon rank-sum test.

^3^Listing of the top 5 phylum in relative abundance of rumen bacteria in lactating dairy cows.

^4^Listing of the top 10 genus in relative abundance of rumen bacteria in lactating dairy cows.

### 3.7 Correlation analysis

In order to comprehensively investigate the potential relationship between cow lactation performance and other key indicators, Mantel-test and Spearman's correlation analysis were used in this study. It is important to note that the Mantel-test evaluates correlations between distance matrices, reflecting structural similarity rather than the directional trends of individual variables. Therefore, the term “significant correlation” is used uniformly for Mantel results, without specifying positive or negative direction, to distinguish it from the directional interpretation in Spearman's correlation analysis.

**Figure 3:**
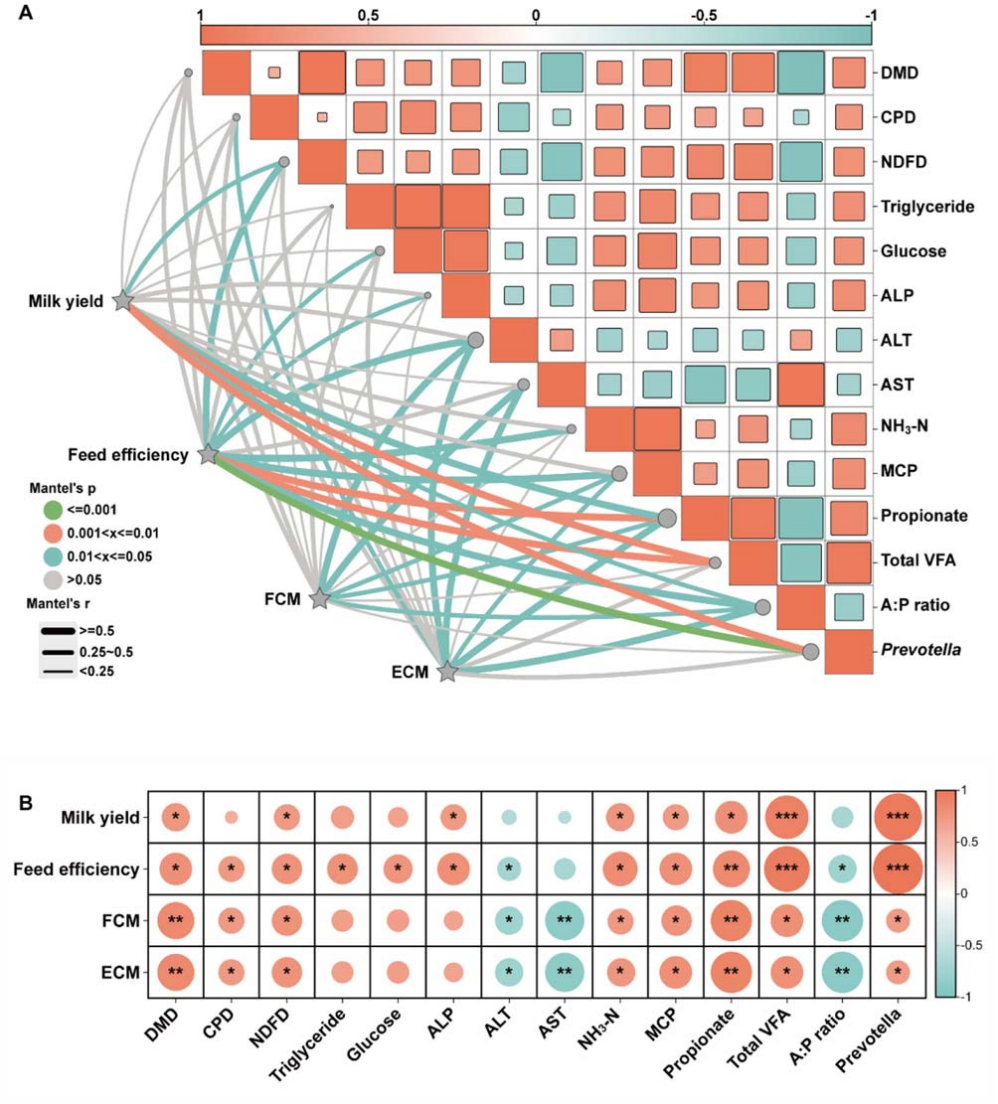
Correlation analysis between lactation performance and other indices in lactating dairy cows. **(A)** Mantel-test correlation analysis. **(B)** Spearman's correlation analysis. FCM, fat corrected milk; ECM, energy corrected milk; DMD, dry matter digestibility; CPD, crude protein digestibility; NDFD, neutral detergent fiber digestibility; ALP, alkaline phosphatase; ALT, alanine aminotransferase; AST, aspartate aminotransferase; NH_3_-N, ammonia-nitrogen; MCP, microbial crude protein; VFA, volatile fatty acids; A:P ratio, acetate-to-propionate ratio. *P < 0.05; **P < 0.01; ***P < 0.001.

As shown in Figure 3A, the Mantel test results showed significant correlations between lactation performance indicators including milk yield, FE, FCM and ECM and rumen fermentation parameters. These indicators were significantly correlated with propionate and A:P ratio (*P* < 0.05); FE, FCM and ECM were significantly correlated with MCP (*P* < 0.05), and FE was also significantly correlated with NH3–N (*P* < 0.05). In addition, milk yield and FE were significantly correlated with TVFA and *Prevotella* (*P* < 0.05). Significant correlations were also found between lactation performance and plasma liver function indicators as FCM and ECM were significantly correlated with ALT and AST (*P* < 0.05), while FE was also correlated with ALT and ALP, respectively (*P* < 0.05). Moreover, FE was significantly correlated with NDFD (*P* < 0.05), and ECM and FE were significantly correlated with CPD and glucose, respectively (*P* < 0.05).

As shown in Figure 3B, the results of Spearman's correlation analysis were highly consistent with Mantel-test, which further validated the above findings. Lactation performance indicators showed significant positive correlations with propionate, MCP, NH3–N, TVFA and *Prevotella* (*P* < 0.05), while significant negative correlations were found with A:P ratio (*P* < 0.05). Additionally, lactation performance was positively correlated with apparent nutrient digestibility of DM, CP and NDF (*P* < 0.05), while FCM and ECM were significantly negatively correlated with ALT and AST (*P* < 0.05), and FE was significantly positively correlated with triglycerides, blood glucose and ALP (*P* < 0.05).

## 4 Discussion

This study evaluated the impact of substituting 5.3% maize with 2.6% MFRB in the diets of lactating Holstein cows over a 30-day period. The results showed a reduction in DMI by 1.02 kg/d, along with significant increases in milk yield, FE, FCM, and ECM. Moreover, cows receiving MFRB demonstrated improved nutrient digestibility and enhanced rumen fermentation, as evidenced by significantly higher NDFD, ruminal propionate, MCP, NH3–N, and *Prevotella* abundance. Additionally, plasma levels of triglycerides and glucose increased, while liver function enzymes, including AST and ALT, exhibited altered activities, indicating reduced metabolic stress on the liver.

The observed increase in milk yield and feed efficiency, despite a reduction in DMI, is partly consistent with the findings of Jiang et al. (27), who reported that replacing soybean meal with a fermented corn gluten–wheat bran mixture improved lactation performance and feed efficiency in Holstein cows, although without affecting DMI. In contrast, the DMI reduction may be attributed to the specific microbial consortia used in MFRB fermentation (*Bacillus licheniformis, Bacillus subtilis, Lactobacillus acidophilus*, and *Saccharomyces cerevisiae*), which likely produced bioactive metabolites such as short-chain fatty acids or peptides that enhanced nutrient utilization or modulated appetite regulation (28). These findings are further supported by Wang et al. (29), who documented that feeding a fermented total mixed ration improved NDFD, DMD and CPD, enhanced ruminal VFAs and milk fat efficiency despite a reduction in DMI. This may be attributed to microbial pretreatment increasing fiber accessibility and enhancing the efficiency of rumen microbial utilization, thereby generating more VFA-rich substrates even with lower feed intake (30, 31). Similarly, Polyorach et al. (32) reported that microbial fermented liquid promoted milk production in early-lactating dairy cows by improving NDFD and CPD, though it was associated with increased DMI. The discrepancy in DMI trends between the two studies may stem from differences in the form, composition, and fermentation degree of the microbial additives, as well as variations in the physiological stages of the animals and the basal diet composition (33). Taken together, these results indicate that the decline in DMI did not impair, but rather coincided with, improved lactation performance. The combination of higher NDFD, elevated ruminal propionate and MCP, and increased CPD suggests that a greater proportion of the ingested nutrients was converted into metabolizable energy and microbial protein available for milk synthesis. Thus, the reduced DMI in cows fed MFRB is more likely to reflect enhanced digestive and metabolic efficiency than a negative response in feed intake or health. Dietary supplementation with MFRB had a significant effect on lactation performance. The elevated milk fat yield likely arises from increased ruminal VFAs which is key substrates for *de novo* fatty acid synthesis in the mammary gland, driven by *Prevotella*-mediated fiber fermentation (34). Coleman et al. (35) findings where Saccharomyces cerevisiae fermentation product supplementation for enhanced milk fat and protein yields alongside FCM and ECM improvements in lactating cows. Moreover, a meta-analysis reported that SC increased 3.5% FCM by 1.6 kg/d and ECM by 1.7 kg/d, further supporting the role of microbial fermentation in optimizing lactogenic nutrient supply (36).

Ruminal NH3–N concentration reflects the balance between dietary protein deamination and microbial utilization, serving as a key determinant of MCP synthesis (37). In this study, MFRB supplementation increased ruminal NH3–N, likely due to enhanced proteolysis associated with *Prevotella* expansion (38). This rise in NH3–N was paralleled by a significant increase in MCP concentration, indicating more efficient nitrogen capture by rumen microbes (39). The augmented MCP supply subsequently elevated CPD, reflecting improved true protein flow to the small intestine for absorption and milk protein synthesis (40). These interrelationships among NH3–N, MCP, and CPD have been similarly reported in dairy diets optimized for rumen degradable protein, where enhanced NH3–N and MCP synthesis improved CPD without compromising cow performance (41). Elevated ruminal propionate levels observed in cows fed MFRB are consistent with previous *in vitro* findings using rice bran fermented with *Ligilactobacillus equi*, which demonstrated increased VFA production (17). In the present study, rumen microorganisms transformed into propionate-type fermentation and the rise in propionate likely contributed to enhanced gluconeogenesis (42), as evidenced by elevated plasma glucose concentrations, thereby supporting increased milk yield and protein synthesis. Increased MCP and amino acid availability in the rumen, which underlie the observed increase in milk protein percentage, together with improved energy status from increased propionate, likely promote the observed increase in milk yield. Notably, MFRB significantly enriched *Prevotella*, a bacterial genera well known for its role in carbohydrate and protein metabolism and its preference for propionate production over methanogenesis (43, 44). Similar shifts in the rumen microbiome were reported by Hu et al. (45), where beef cattle fed rice straw co-fermented with probiotics showed increased abundance of Bacteroidetes genera, improved production of propionate and acetate. The increased *Prevotella* abundance observed in this study may thus represent a key mechanistic pathway underlying improved rumen fermentation, aligning with its established role in redirecting rumen carbon into host energy metabolism (46).

Dietary supplementation with MFRB had a significant increased in plasma glucose and triglycerides. The surge in ruminal propionate production likely fueled hepatic gluconeogenesis, driving the increase in circulating glucose, while improved rumen MCP and VFA synthesis provided more precursors for very-low-density lipoprotein assembly and export, thereby raising triglyceride and glucose levels. The same effect mirrored by postpartum feeding of fermented ammoniated corn fiber, which boosted both plasma glucose in early lactation cows (47). Concurrently, plasma ALT and AST activities declined significantly, indicating reduced hepatocellular stress and enhanced liver integrity (48). Similar hepatic benefits have been observed when dry-off and peripartum cows received Saccharomyces cerevisiae fermentation products, which lowered transaminase levels and supported antioxidant defenses (49). In contrast, plasma ALP levels were upregulated, which may be related to MFRB induced enhancement of hepatic membrane-associated metabolic activity or changes in biliary excretory function, and the changes may reflect an adaptive response to the regulation of fermentation product or mineral metabolism (50). These shifts in energy substrates and liver function underpin the improved milk yield, FCM, and ECM seen with MFRB, highlighting the pivotal role of optimized rumen to liver integration in dairy cow lactation performance (51). From a production standpoint, the simultaneous reduction in DMI and increase in milk, FCM, and ECM yields clearly demonstrates an improvement in FE in cows receiving MFRB. Improving FE while slightly lowering DMI is advantageous under current conditions of high feed costs and the need to reduce reliance on human-edible grains, provided that cow health and metabolic status are maintained. In the present study, the higher plasma glucose and triglyceride concentrations, together with the reduced ALT and AST activities, suggest that cows fed MFRB did not experience aggravated negative energy balance or hepatic stress despite the lower DMI. Instead, the data support a scenario in which microbial fermentation of rice bran enhances the efficiency of rumen–liver nutrient utilization, allowing cows to sustain greater lactation output with a smaller intake of feed.

Correlation analyses reinforced a microbiota—host—performance axis: lactation performance indicators were strongly tied to ruminal propionate, MCP, NH3–N, TVFA, and *Prevotella* abundance. *Prevotella* promotes hepatic gluconeogenesis and plasma glucose by altering rumen VFA production, which in turn supports improved lactation performance and milk composition. Concurrent associations with ALT and AST suggest that MFRB also modulates hepatic metabolism, potentially through microbial metabolites or altered nutrient flux. These observed biochemical changes emphasize the critical role of microbiota–host interactions in regulating metabolic efficiency and productivity (52). Overall, these findings highlight that the MFRB-induced reduction in DMI is accompanied by more efficient nutrient capture and partitioning toward milk, rather than by a detrimental restriction in nutrient supply. Milk composition thus emerges as a highly integrated process, involving cross-system coordination among rumen microbial metabolism, plasma biochemical homeostasis, and hepatic nutrient processing within a multi-layered physiological regulatory network (53–55). However, this study's limitation is the lack of multi-omics analyses to explore lactation performance changes from multiple mechanistic angles. Integrating metabolomics, metagenomics, and transcriptomics will provide a more comprehensive view of how MFRB modulates rumen–host interactions. Future research will focus on applying these multi-omics techniques to unravel the molecular mechanisms underlying the observed improvements in dairy cow productivity.

## 5 Conclusion

Dietary supplementation with 2.6% MFRB in lactating Holstein cows resulted in enhancements in milk yield, FE, FCM and ECM, and NDFD, despite a reduction in DMI. These benefits were underpinned by shifts in rumen fermentation, particularly increased propionate, MCP, NH_3_-N and *Prevotella* abundance, as well as improved plasma triglycerides, glucose and decreased in AST and ALT. Therefore, MFRB is a sustainable feed that enhances nutrient utilization and dairy cow production, aligning with circular dairy practices.

## Data Availability

The datasets analyzed during the current study are not publicly available due to confidentiality and proprietary restrictions related to the project. Access to the data may be granted by the corresponding author upon reasonable request, subject to approval by the data owner.
